# The Impact of the Aging Workforce on Occupational Medicine: A Narrative Review

**DOI:** 10.7759/cureus.113037

**Published:** 2026-07-20

**Authors:** Joshua A Jogie, Rondon Ramlal, Mitra Maharaj, Kevin Maraj, Mahendra Singh, Kenisha Phillip, Donna Rampersad

**Affiliations:** 1 Occupational Health Unit, St. James Medical Complex, Port of Spain, TTO; 2 Industrial Medicine Unit, Eric Williams Medical Sciences Complex, Champs Fleurs, TTO; 3 Occupational Medicine Unit, San Fernando General Hospital, San Fernando, TTO; 4 Internal Medicine, St. James Medical Complex, Port of Spain, TTO

**Keywords:** aging workforce, occupational health, occupational medicine, older workers, work ability

## Abstract

Population aging is changing the workforce and the practice of occupational medicine. Older workers bring experience, judgment, and knowledge. But they may also have wider differences in physical reserve, sensory function, sleep tolerance, chronic disease, and recovery after illness or injury. This review examines the effects of these changes on work ability, fitness-for-work assessment, injury prevention, health surveillance, sickness absence, return to work, and workplace accommodation.

We conducted a narrative review of PubMed-indexed, English-language human studies from database inception to June 2026. The search covered aging workers, occupational medicine, work ability, physical and cognitive capacity, chronic disease, work injury, shift work, workplace accommodation, return to work, and age management. We included 46 publications after screening for relevance. We grouped the evidence by theme. We did not perform a meta-analysis or formal risk-of-bias assessment.

The evidence supports assessment based on function, job demands, hazards, and available controls rather than age alone. The strongest evidence supports broad principles. These include reducing the mismatch between worker capacity and job demands, improving ergonomic and fatigue controls, supporting chronic disease management, and using individual, time-limited accommodations. Other important issues include multimorbidity, polypharmacy, frailty, sarcopenia, burnout, ageism, organizational justice, and digital adaptation. But the intervention evidence is varied and limited by observational study designs, inconsistent definitions, and the healthy worker effect.

Occupational medicine is moving from one-time medical clearance to long-term management of work ability. Occupational physicians should combine clinical findings with job analysis, prevention, rehabilitation, and organizational advice. Sustainable work needs age-inclusive design, fair access to training and accommodation, and regular review of risk controls.

## Introduction and background

Population aging is changing the workforce in many countries [1]. More adults are working beyond the usual retirement age because people live longer, need income, face pension reform, or work in sectors with staff shortages [1,2]. This change affects more than workforce numbers. It changes the cases, decisions, and services seen in occupational medicine. Occupational physicians now manage more chronic disease, functional variation, medication effects, repeated absence, and complex return-to-work decisions across longer working lives [2,3].

Older workers are not one uniform group. Age alone does not predict health, safety, productivity, or fitness for work [3,4]. Some older workers retain high physical and cognitive capacity. Others develop reduced strength, pain, sensory loss, fatigue, or slower recovery [4,5]. The main question is not whether a worker is old. It is whether the worker's current function matches the essential demands and hazards of the job, and whether suitable controls can reduce risk [3,6].

An aging workforce also brings clear benefits. Older workers may provide experience, technical knowledge, reliability, judgment, and knowledge of workplace systems [2,7]. Age management should preserve these strengths while reducing avoidable risk through ergonomic design, flexible work, training, and early occupational health support [8]. This requires a move from one-time medical clearance to long-term work-ability management.

Work ability is the balance between a worker's health, function, skills, values, and job demands [3,9]. Job demands may be physical, cognitive, psychosocial, environmental, or safety-critical [9,10]. Ilmarinen and Rantanen described the promotion of work ability during aging as a main occupational health goal [11]. Later evidence showed that work ability depends on both personal and workplace resources. Workplace resources include job control, supervisor support, fairness, training, and ergonomic design [9,12]. Reduced work ability is therefore not always due to disease. It may also result from poor job design or workplace conditions that can be changed.

Aging may reduce aerobic capacity, muscle strength, flexibility, balance, heat tolerance, and recovery after effort [4,5]. These changes matter most when a worker's reserve is close to the demands of heavy lifting, climbing, prolonged standing, repetitive work, emergency response, or heat exposure. Injury risk rises when physical ability does not match job demands [10]. Back pain, osteoarthritis, neck and shoulder disorders, and cumulative tendon injury may further reduce work ability and delay recovery [13,14].

Sensory changes may affect visual acuity, contrast sensitivity, glare tolerance, depth perception, hearing, balance, and night vision [15-17]. These changes may affect driving, machinery use, alarm recognition, work at height, and night work. Aging may also reduce respiratory reserve. Cardiovascular disease, hypertension, arrhythmia, and diabetes may affect heavy work and safety-critical duties [18,19]. These diagnoses do not by themselves mean that a worker is unfit. The assessment should consider disease control, symptoms, complications, medication effects, exposure, and the likely harm from sudden incapacity.

Chronic disease management is now a major part of occupational medicine. Workers with hypertension, diabetes, arthritis, respiratory disease, cancer survivorship, chronic kidney disease, mental health conditions, or chronic pain may remain productive when adjustments are timely and specific [6,20]. Shift work adds another challenge because age may affect sleep, circadian tolerance, recovery, medication timing, and commuting safety [21]. Cognitive aging also needs balanced assessment. Processing speed and divided attention may decline, while vocabulary, practical judgment, pattern recognition, and experience may remain strong [22].

Workplace health promotion and age-friendly measures may support health and continued work, but programs differ in design and effect [23,24]. Earlier reviews often focus on one area, such as work ability, health promotion, shift work, or accommodation. Few combine clinical assessment, workplace controls, multimorbidity, fatigue, psychosocial risk, digital adaptation, and return to work in one occupational medicine model. This gap matters because occupational medicine must now link prevention, clinical risk assessment, rehabilitation, and fair employment decisions instead of relying on age-based clearance.

This review has four objectives. First, it examines how workforce aging is changing occupational medicine. Second, it summarizes evidence on work ability, physical and cognitive capacity, chronic disease, injury, fatigue, and psychosocial risk. Third, it describes practical approaches to fitness-for-work assessment, surveillance, accommodation, and return to work. Fourth, it identifies limits in the evidence and priorities for research. The review covers adults in occupational settings and focuses on evidence that is relevant to clinical and workplace practice.

To the authors' knowledge, this is the first publication from Trinidad and Tobago involving all occupational medicine departments across the country's Regional Health Authorities.

## Review

Materials and methods

This article is a narrative review, not a systematic review or meta-analysis. We searched PubMed from database inception to June 2026. Search terms included “aging workforce,” “older workers,” “occupational medicine,” “occupational health,” “work ability,” “Work Ability Index,” “workplace accommodation,” “chronic disease,” “multimorbidity,” “return to work,” “work injury,” “physical capacity,” “cognitive aging,” “shift work,” “fatigue,” “age management,” and related terms.

We included English-language, PubMed-indexed human studies involving adults. Eligible papers addressed older workers, occupational health practice, work ability, functional capacity, workplace injury, chronic disease at work, shift work, psychosocial factors, workplace interventions, accommodation, retirement, or return to work. We considered reviews, cohort and population studies, qualitative studies, and occupational medicine papers with clear practical relevance. We excluded animal studies, pediatric studies, non-English papers, papers without a workplace or occupational health focus, and papers that did not address the review themes.

We screened titles and abstracts for relevance and reviewed full texts when needed. We selected references by theme, as expected for a narrative review. We included 46 publications. We grouped the evidence into work ability, physical and sensory capacity, chronic disease and multimorbidity, injury and return to work, fatigue and shift work, psychosocial and organizational factors, workplace accommodation, and age-friendly work design.

We did not perform a meta-analysis because the studies used different populations, exposures, outcomes, and definitions. We did not use a formal risk-of-bias tool because the aim was a clinical narrative synthesis rather than a pooled estimate. We improved rigor by separating systematic reviews from observational and qualitative studies, comparing areas of agreement and uncertainty, and avoiding causal claims from observational evidence. The findings are a structured narrative synthesis, not a complete systematic list of all available studies.

Occupational medicine and the changing workforce

The aging workforce is changing occupational medicine. The specialty is moving from one-time medical clearance to long-term management of work ability. Systematic reviews of interventions for aging workers show mixed effects on work ability, productivity, and early retirement. This is partly because the interventions, settings, and outcomes differ [25,26]. The reviews agree that individual support and workplace change matter, but they do not identify one program that works in every setting.

Work in later life can support income, identity, social contact, and mental health. But long exposure to unsafe or poorly designed work can harm health [27]. Occupational medicine should preserve beneficial work and change harmful work. This requires clinical assessment, job analysis, prevention, rehabilitation, and organizational advice.

Work Ability Index and functional assessment

Flexible work and a supportive workplace are linked with fewer health-related work limits in older workers [28]. Chronic disease may increase interest in early retirement when job demands become hard to sustain [29]. Physical job strain may interact with cognition and performance in later life [30]. In contrast, work with cognitive and social engagement may be linked with better cognitive function [31]. These findings support assessment of the fit between the worker and the job rather than the diagnosis alone.

The Work Ability Index (WAI) is a structured occupational health tool. It covers current work ability compared with lifetime best, work ability in relation to job demands, diagnosed diseases, estimated work impairment, sickness absence, expected future work ability, and mental resources. Scores range from 7 to 49. They are often grouped as poor (7-27), moderate (28-36), good (37-43), or excellent (44-49). The WAI can support surveillance, early intervention, follow-up, and discussion of health and job demands [3,9,11]. But it is not a stand-alone fitness certificate. It does not replace clinical assessment, job analysis, or review of safety-critical risk. A low score should lead to assessment of factors that can be changed, not automatic exclusion from work.

**Figure 1:**
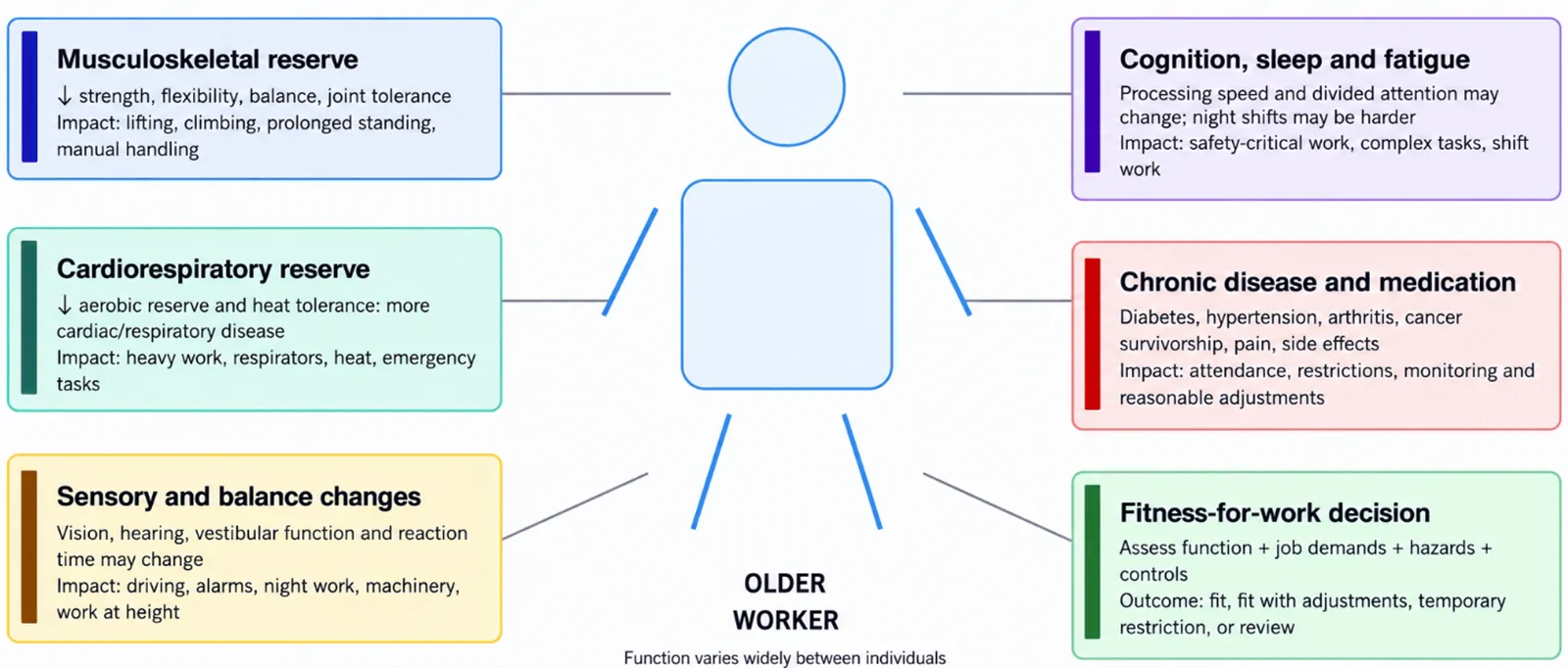
Age-Related Changes and Their Impact on Fitness-for-Work Assessment in Older Workers Age-related changes may affect musculoskeletal, cardiorespiratory, sensory, cognitive, and chronic disease domains. Fitness-for-work decisions should be based on function, job demands, hazards, and controls rather than age alone. Figure created in Microsoft PowerPoint (Microsoft Corporation, Redmond, WA, USA) using native shapes, text boxes, and line work.

Functional assessment should define the essential tasks, how often and how long they are done, physical and cognitive demands, environmental hazards, fatigue, commuting, and the effect of error or sudden incapacity. The clinical assessment may include strength, endurance, balance, mobility, vision, hearing, cognition, disease control, medication effects, and recovery. The outcome may be fit, fit with adjustments, temporarily restricted, or unfit pending further assessment. Restrictions should be specific, proportionate, time-limited when possible, and reviewed. The main age-related changes that may affect fitness-for-work decisions are summarized in Figure 1.

Shift work, fatigue, and fatigue risk management systems

Shift work has been linked with cognitive impairment in middle-aged and older adults [32]. Reviews also link shift work and long hours with reduced attention, memory, and response inhibition [33]. These findings matter most in health care, transport, manufacturing, security, and emergency work, where fatigue may harm the worker and the public. Most evidence is observational. It supports fatigue controls, not general age-based restrictions.

Ergonomic scheduling may protect sleep in aging shift workers [34]. A fatigue risk management system (FRMS) applies this evidence through workplace controls. Main elements include leadership responsibility, identification of fatigue hazards, review of rosters and overtime, limits on consecutive night shifts and short rest periods, forward rotation when practical, protected recovery time, worker reporting without punishment, education on sleep and medication effects, and monitoring of fatigue-related incidents and near misses. Safety-critical work also needs clear escalation, temporary task changes, and regular review of controls. Fatigue management should not depend only on personal resilience or caffeine. It should improve the design of work.

Psychosocial risk, burnout, ageism, organizational justice, and digital adaptation

Workplace programs to reduce stress in older workers may help, but the evidence is limited and varied [35]. Many workers with health limits do not receive needed accommodations. This may increase absence, reduce productivity, and lead to early exit from work [36]. Longer working lives may support health when work is safe, meaningful, and flexible. They may cause harm when poor work is simply continued for longer [37].

Burnout may occur when high workload, low control, emotional demand, poor staffing, or long-term role conflict causes exhaustion and reduced work performance. Older workers are not automatically at greater risk. But they may have longer exposure to poorly controlled stress or may also manage caregiving and health problems. Assessment should separate burnout-related impairment from depression, sleep disorders, medication effects, and physical illness.

Ageism is the use of stereotypes, such as the belief that older workers are slow, inflexible, resistant to technology, or unable to learn. These views may reduce access to training, promotion, varied work, and accommodation. Organizational justice means fair procedures, respectful treatment, clear decisions, and equal access to resources. It supports trust and work ability. Occupational physicians should not reinforce age bias. Their advice should be based on documented function and risk.

Digital adaptation is now an occupational health issue because workplaces use electronic records, automation, remote systems, and artificial intelligence. Difficulty with these systems may result from poor design, weak training, high visual demands, or rushed introduction rather than age. Useful controls include paced training, accessible interfaces, suitable font size and contrast, protected learning time, peer support, and assessment based on competence. Digital change is a work-design issue, not proof of age-related unfitness.

Physical work, multimorbidity, frailty, sarcopenia, and polypharmacy

The link between physically demanding work and disability in later life is complex. It is affected by health selection, survival, social and economic conditions, and lifestyle [38]. Continued paid work among people with chronic disease depends on health, personal resources, and job features [39,40]. Early retirement also reflects health, financial, and organizational factors [41]. Chronic disease may reduce work capacity, but early support may help workers remain employed [42].

Older workers often have several diseases rather than one diagnosis. These conditions may interact and increase fatigue, pain, mobility limits, hypoglycemia risk, cognitive load, or sensitivity to heat and shift work. Frailty is reduced physiological reserve and greater vulnerability to stress. Sarcopenia is loss of muscle mass and strength. Neither should be assumed from age alone. Relevant signs include weakness, slow walking, repeated falls, weight loss, low activity, and poor recovery. When needed, assessment may include gait, chair rise, grip strength, balance, endurance, nutrition, and recent changes in function.

Polypharmacy may affect work through sedation, postural symptoms, bleeding risk, hypoglycemia, urinary frequency, impaired heat control, or complex dosing. Occupational assessment should consider the combined effects of medicines, timing in relation to shifts, adherence, and the harm that impaired performance could cause. Medication review should be coordinated with the treating clinician. Occupational physicians should not change treatment only to meet work demands without a clinical reason.

Chronic disease, return to work, and work retention

Systematic evidence shows that work participation with chronic disease depends on disease factors, personal factors, job demands, and social support [43]. Heavy physical work, low control, poor support, and psychosocial strain may lead to earlier retirement [44]. Qualitative evidence also shows that work may support identity, routine, self-management, and independence in older workers with several chronic diseases [45]. The worker's view of their health is also important. A worker who feels unable to cope may need rehabilitation, reassurance, or adjustment rather than simple clearance [46].

A return-to-work plan should state the hours, duties, prohibited exposures, expected duration, and review date. Measures may include shorter hours, graded workload, temporary removal from night work or safety-critical tasks, ergonomic changes, less travel, rehabilitation, and planned clinical review. The plan should use the least restrictive safe option. Permanent exclusion should usually follow adequate treatment, rehabilitation, job analysis, and review of possible controls.

Health surveillance should be based on exposure and evidence. Age alone is not a reason for extra surveillance. But earlier review of functional change may be needed for respirator use, noise exposure, driving, work at height, heat, hazardous substances, or emergency response. Surveillance should lead to prevention and control, not discriminatory removal from work.

Evidence synthesis and occupational medicine implications

Table 1 summarizes the main evidence, areas of agreement and uncertainty, strength of evidence, and practical implications for occupational medicine.

**Table 1:** Critical Synthesis of Evidence and Occupational Medicine Implications

Domain	Main evidence	Agreement/uncertainty	Evidence base/strength	Occupational medicine implication
Work ability	Function depends on health, demands, flexibility, and support [25,28,29].	Broad agreement; intervention effects vary.	Systematic reviews plus cohort evidence; moderate for principles, low-to-moderate for specific programs.	Use Work Ability Index as a screening and monitoring aid, then complete job-specific assessment.
Physical capacity and injury	Mismatch between physical ability and job demands increases risk [10,13,14,30].	Agreement on mismatch; individual decline is highly variable.	Observational studies and reviews; moderate.	Assess actual tasks and introduce ergonomic controls, aids, pacing, and phased duties.
Shift work and fatigue	Shift work and long hours may affect sleep and cognition [32-34].	Association is consistent, but causality and age thresholds are uncertain.	Observational studies and review evidence; moderate for fatigue control, low for age cut-offs.	Use a fatigue risk management system and individualized assessment rather than blanket exclusion.
Chronic disease and multimorbidity	Work participation reflects disease, personal, and work factors [39,42-46].	Strong agreement that diagnosis alone is insufficient.	Systematic review, cohorts, and qualitative evidence; moderate.	Review interactions, medications, functional reserve, and feasible adjustments.
Psychosocial and organizational factors	Stress, accommodation, flexibility, and organizational climate influence retention [28,35-37,44].	Agreement on importance; intervention studies are heterogeneous.	Systematic reviews and cohorts; low-to-moderate.	Address burnout, ageism, fairness, job control, and access to training.
Accommodation and return to work	Specific accommodations can close the worker-job gap [20,36,43].	Agreement on individualized support; limited comparative evidence on optimal packages.	Systematic review and observational evidence; moderate for principle.	Give clear, proportionate, time-limited advice with planned review.

Discussion

This review combines clinical, functional, and organizational evidence in one occupational medicine framework. Earlier reviews showed that work ability depends on personal and workplace resources and that age-management programs have mixed effects [9,12,25,26]. This review agrees with those findings. It also gives more attention to how occupational physicians can apply them to fitness-for-work decisions, fatigue controls, chronic disease management, rehabilitation, and workplace advice.

The most consistent finding is that age alone is a poor measure of function. The studies use different groups and outcomes, but they repeatedly show that work participation depends on the interaction between health and job demands [10,28,39,43]. The evidence also supports flexibility, supportive management, and accommodation as ways to preserve employment [20,28,36]. But the evidence is stronger for these broad principles than for any single intervention program.

Important uncertainties remain. Observational studies cannot fully separate aging from cumulative exposure, social and economic conditions, or health selection. Some studies suggest that demanding work may reduce later function, while others do not show a simple direct link [30,38]. Shift-work studies support fatigue controls but not one age limit for all workers [32-34]. Studies of longer working lives also show that work may help or harm health depending on job quality and the worker's circumstances [27,37]. These differences support individual assessment and better intervention research.

This review also identifies gaps that are often separated from older-worker research. Multimorbidity, frailty, sarcopenia, and polypharmacy may combine to reduce reserve even when each condition appears stable. Burnout, ageism, unfair workplace decisions, and poor access to digital training may also reduce work ability without causing a clear disease. Future studies should measure these factors as main issues, not minor background variables.

Occupational medicine needs new service models because periodic clearance does not reflect the changing relationship between health and work. A better model combines surveillance, early referral, job-demand analysis, the WAI or similar screening tools, fatigue management, ergonomic review, chronic disease support, and planned follow-up. The occupational physician should connect clinical care with workplace prevention while protecting confidentiality and avoiding unnecessary disclosure.

Future studies should use consistent definitions of older worker, work ability, sustainable employment, and successful return to work. Prospective studies should report job demands, multimorbidity, medication burden, psychosocial conditions, and accommodation. Trials should compare practical intervention packages. They should measure safety, quality of life, productivity, fairness, and lasting work participation, not only retirement or absence. Evidence from low- and middle-income countries and informal workplaces remains limited.

Role of the occupational physician

The occupational physician should link medical findings to essential job demands and possible harm. Advice to the employer should state the worker's capability, restrictions, adjustments, likely duration, and review date. It should not disclose unnecessary clinical details. Records should include relevant hazards, symptoms, functional findings, medication effects, risk of sudden incapacity, and the controls considered.

When risk is uncertain, temporary restriction with planned reassessment may be safer and fairer than permanent exclusion. When failure could cause serious harm, temporary removal from safety-critical work may be needed during investigation or treatment. Prevention advice should cover physical load, workstation design, lighting, noise, fatigue, staffing, training, psychological safety, and early reporting. These controls help workers of all ages.

Limitations

This narrative review does not provide pooled effect estimates or a formal risk-of-bias score. We report the search period, eligibility criteria, and selection process, but the review was not designed to be as exhaustive or reproducible as a systematic review. We included only PubMed-indexed English-language papers. This may have excluded useful policy documents, non-indexed evidence, and studies in other languages.

The studies use different definitions of older worker, work ability, health limitation, and successful aging at work. Many are observational and may be affected by confounding and the healthy worker effect. Workers who remain employed are often healthier than those who have left work. Studies based in workplaces may therefore miss people who stopped working because of illness.

There are fewer intervention studies, and they differ in design, population, outcome, and follow-up. The evidence is therefore stronger for broad principles than for one fixed program. The findings may not apply equally across countries because laws, pension systems, health care access, workplace culture, and job demands differ. Advice must be adapted to local law, policy, and the actual workplace.

## Conclusions

The aging workforce is moving occupational medicine away from age-based clearance and toward long-term management of work ability. Older workers bring skill, judgment, experience, and stability. But they also show wide differences in physical reserve, sensory function, sleep tolerance, multimorbidity, medication burden, and recovery. Age alone should not decide fitness for work. The best approach links clinical function with essential job demands, hazards, possible harm, and available controls. Occupational physicians should use structured work-ability assessment, ergonomic and fatigue controls, chronic disease and medication review, specific accommodations, and planned return-to-work follow-up. They should also address burnout, ageism, organizational justice, and barriers to digital adaptation. The evidence supports these broad principles, but it is varied and often observational. Better prospective and intervention studies are needed, especially on multimorbidity, frailty, sustainable shift work, digital change, and outcomes in different economies. The goal is not only longer employment. It is safe, fair, productive, and sustainable work across the life course.
